# COVID-19 mortality in the Philippines: province-level ecological analysis, 2020–2023

**DOI:** 10.5365/wpsar.2026.17.1.1128

**Published:** 2026-03-25

**Authors:** Jimuel Celeste, Jesus Emmanuel Sevilleja, Vena Pearl Bongolan, Roselle Leah Rivera, Salvador Eugenio Caoili, Romulo de Castro

**Affiliations:** a System Modeling and Simulation Laboratory, Department of Computer Science, College of Engineering, University of the Philippines Diliman, Quezon City, Philippines.; b Center for Informatics, University of San Agustin, Iloilo City, Philippines.; c Mental Health Research Unit, Office for Special Concerns, National Center for Mental Health, Mandaluyong City, Philippines.; d Hospital Epidemiology Surveillance Unit, Public Health Unit, National Center for Mental Health, Mandaluyong City, Philippines.; e Department of Women and Development Studies, College of Social Work and Community Development, University of the Philippines Diliman, Quezon City, Philippines.; f Biomedical Innovations Research for Translational Health Science (BIRTHS) Laboratory, Department of Biochemistry and Molecular Biology, College of Medicine, University of the Philippines Manila, Manila, Philippines.; g Health Informatics Program, Institute of Health Sciences and Nursing, Far Eastern University, Manila, Philippines.

## Abstract

**Objective:**

To investigate COVID-19 mortality in Philippine provinces from 2020 to 2023.

**Methods:**

Crude mortality rate (CMR), age-standardized mortality rate (ASMR) and age-specific mortality rate were computed for 84 areas (82 provinces and 2 cities) using COVID-19 surveillance data from the Philippine Department of Health, which captured data about confirmed deaths occurring between 20 January 2020 and 9 May 2023. Provinces were ranked by their ASMR. A correlation analysis was conducted to identify possible predictors of COVID-19 mortality. Among the factors investigated were the incidence of poverty, population density, proportion of the population considered elderly (aged ≥ 65 years), hospital bed density and COVID-19 testing rates.

**Results:**

Eight of the 10 provinces that had the highest COVID-19 ASMRs were located in the Luzon island group. The province with the highest ASMR was Benguet in Northern Luzon (207.83 deaths/100 000 population), and the lowest rate was in Tawi-Tawi in Southwestern Mindanao (2.22 deaths/100 000 population). The incidence of poverty was negatively correlated with COVID-19 mortality, while hospital bed density and COVID-19 testing rates were positively correlated with CMRs and ASMRs.

**Discussion:**

This analysis provides a starting point for investigating COVID-19 mortality in Philippine provinces. The ranking of provinces by their ASMR is useful for directing future epidemiological investigations and, coupled with the results of the correlation analysis, provides insight into the factors that may have impacted COVID-19 mortality in the Philippines. Our analysis suggests that COVID-19 mortality patterns can partly be explained by the streetlight effect and factors linked to the availability of and access to health care.

The COVID-19 pandemic caused millions of deaths globally. As of 29 December 2024, the cumulative total number of COVID-19 deaths reported to the World Health Organization (WHO) stood at just over 7.07 million. (*1*) The corresponding figure for the Philippines was 66 800 deaths. (*1*, *2*) Although the numbers of cases and deaths are not as high as they were when COVID-19 was a public health emergency of international concern, it is important to investigate what transpired during the pandemic, to look back and reflect, and to learn from the experience, given the risk of the emergence of new viruses. (*3*, *4*)

As a first step towards this goal, this study undertook a provincial-level investigation of COVID-19 mortality in the Philippines. The aims of the analysis were, first, to identify the provinces with the highest and lowest mortality rates, and, second, to identify possible drivers of high mortality rates. The study’s objective was to add nuance to the understanding of COVID-19 mortality in the Philippines at the provincial level and to provide data to guide future epidemiological investigations and to inform future planning for pandemic preparedness and response, including surveillance.

## Methods

### Data sources

#### COVID-19 data

Data about confirmed deaths from COVID-19 compiled by the Department of Health’s COVID-19 Case Tracker up until 27 May 2023 were analysed. (*2*) This data set includes records of deaths of patients who were diagnosed with COVID-19 as confirmed by reverse transcription–polymerase chain reaction (RT–PCR) conducted by a certified laboratory. (*2*) The first confirmatory test for COVID-19 in the Philippines using RT–PCR was performed at the Research Institute for Tropical Medicine on 20 January 2020. (*5*) The latest death in the data set was recorded on 9 May 2023.

Records were excluded if they did not contain data on the age, sex or province of the person tested. For records missing the date of infection, one of the following variables was used as a substitute, whichever had the first non-missing value: the date of disease onset, specimen collection, result release, report confirmation (i.e. the date when the Department of Health confirmed that the record was accurate) or death. Finally, case data were stratified by 84 administrative areas (82 provinces and 2 cities, the City of Isabela and Cotabato City) and 5-year age groups (0–4, 5–9, … ≥ 80 years).

#### Predictor variables

Possible predictors of COVID-19 mortality were selected depending on the availability of reliable provincial-level data from official government sources and a priori knowledge, with the latest year for the data also noted:

- population density (number of persons/km^2^) (2020); (*6*)
- percentage of the population aged ≥ 65 years (2020); (*7*)
- incidence of poverty (2021); (*8*)
- hospital bed density (number of beds/100 000 population) (2022); (*7*, *9*) and
- COVID-19 testing rates (number of tests performed/100 000 population) (2020–2023). (*7*, *10*)

All of the predictors have been identified as possible determinants of the risk of mortality from COVID-19 in studies conducted in other countries. (*11*-*15*)

Population data and information about the incidence of poverty were from publicly available data sets from the Philippine Statistics Authority. (*6*-*8*) The Authority defines poverty incidence as “the proportion of Filipinos whose per capita income cannot sufficiently meet [their] individual basic food and non-food needs.” (*8*) In 2021, this threshold was set at 12 030 Philippine pesos per month for a family of five. (*8*)

Data on the number of hospital beds and COVID-19 tests were extracted from publicly available data sets curated by the Department of Health. (*2*, *9*, *10*) Hospital bed density was computed as the number of hospital beds divided by the total population of the province, and multiplied by 100 000. (*7*, *9*) Similarly, the COVID-19 testing rate was computed as the number of COVID-19 RT–PCR tests performed divided by the total population of the province, multiplied by 100 000. (*7*, *10*) As the last recorded death occurred on 9 May 2023, RT–PCR testing data were also truncated at this date.

The number of COVID-19 tests performed by each province was estimated from the number of COVID-19 tests performed in all facilities in that province. Data about COVID-19 testing were available by facility, not by province, and people living in one province may have been tested in a neighbouring province. In such cases, their test would be counted in the total of the province where they were tested, not where they lived.

### Statistical analysis

#### Mortality rates

The crude mortality rate (CMR), age-standardized mortality rate (ASMR) and age-specific mortality rate were calculated for each of the 84 administrative reporting units. The ASMR was computed through direct standardization to eliminate the confounding effect of age (i.e. the risk of COVID-19 mortality increases sharply with age) and to facilitate comparisons between provinces with varying age structures, in line with standard practice in global ranking studies. (*11*, *16*)

The following formulas were used to calculate the CMR and ASMR:

 (Equation 1) and

(Equation 2),

where *i* is the age group, *m* is the number of deaths, *p* is the provincial population, *m****/****p* is the age-specific mortality rate and *s* is the standard population. In this study, the standard population was the 2020 population. (*7*) Note that the age-specific mortality rate is different from the ASMR. The age-specific mortality rate is mortality in a specific age group, while the ASMR is calculated using the age-specific mortality rates from all age groups. The ASMR was used to rank the provinces from highest to lowest COVID-19 mortality.

#### Correlation analysis

A correlation analysis was performed to identify possible predictors of COVID-19 mortality and to investigate the relationships between selected possible predictors. Prior to the correlation analysis, the distributions of the CMR, ASMR and the five risk factor variables were explored. On the basis of this analysis, which revealed the presence of non-normal data distributions (**Supplementary Fig. 1**), Spearman’s method was chosen to measure the strength of the relationship between pairs of variables. Spearman’s correlation coefficient (*r*_s_) is considered to be more robust to outliers than Pearson’s correlation coefficient. (*17*) Mukaka’s approach was adopted to aid in interpreting the correlation coefficients: (*17*) *r*_s_ values from −0.30 to 0.00 or 0.00 to 0.30 imply no or a negligible correlation; values from −0.50 to −0.30 or 0.30 to 0.50 imply a weak correlation; values from −0.70 to −0.50 or 0.50 to 0.70 imply a moderate correlation; values from −0.90 to −0.70 or 0.70 to 0.90 imply a strong correlation; and values from −1.00 to −0.90 or 0.90 to 1.00 imply a very strong or perfect correlation. Finally, *P*-values were calculated to test the statistical significance of the correlations found.

**Fig. 1:**
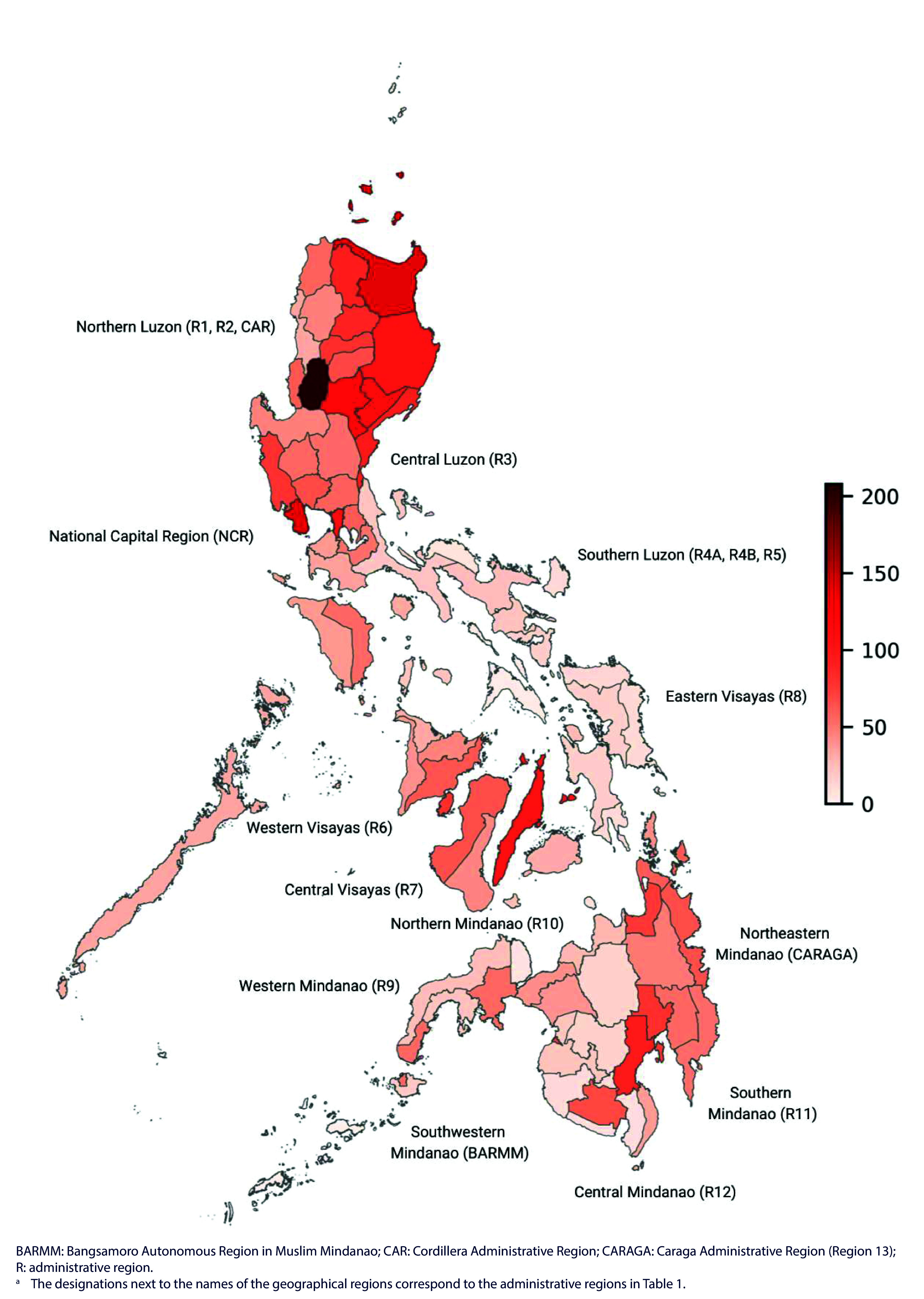
Heatmap of province-level, age-standardized COVID-19 mortality per 100 000 population, Philippines, January 2020–May 2023^a^

[**insert** Figure 1]



Statistical analyses were conducted using Python Pandas software for the correlation coefficients and SciPy for the *P*-values. (*18*-*20*)

## Results

### Crude versus age-standardized mortality

The CMRs and ASMRs for the 84 administrative areas are reported in **Table 1** and **Supplementary Table 1**; also shown are the absolute differences between the CMRs and ASMRs. The median of the absolute difference is 1.26 deaths/100 000 population (interquartile range [IQR]: −2.35, 7.22). Compared with the ASMRs, the CMRs had a higher median value (48.36 vs 44.73 deaths/100 000 population, respectively) and a wider dispersion (IQR: 22.32, 73.08 vs 24.78, 67.48, respectively). These results confirm the presence of the confounding effect of population age on the CMRs. Thus, ranking the provinces by their CMR would be misleading, as this measure does not account for differences in the age structure of the provinces. For this purpose, the ASMR is more appropriate. (*16*)

**Table 1:** Crude and age-standardized COVID-19 mortality per 100 000 population, by province, Philippines, January 2020–May 2023^a^

Province (geographical region/administrative region)^b^	Mortality		Difference (CMR – ASMR)
	CMR	ASMR	
**Benguet (Northern Luzon/CAR)**	**211.03**	**207.83**	**3.20**
**Cagayan (Northern Luzon/R2)**	**163.17**	**139.28**	**23.90**
**Bataan (Central Luzon/R3)**	**136.30**	**137.88**	**-1.58**
**Nueva Vizcaya (Northern Luzon/R2)**	**132.31**	**121.07**	**11.24**
**Quirino (Northern Luzon/R2)**	**118.90**	**115.12**	**3.78**
**National Capital Region (NCR)**	**102.67**	**109.92**	**-7.25**
**Isabela (Northern Luzon/R2)**	**116.72**	**108.25**	**8.47**
**Cebu (Central Visayas/R7)**	**102.90**	**107.51**	**-4.61**
**Aurora (Central Luzon/R3)**	**98.30**	**97.25**	**1.05**
**Davao del Sur (Southern Mindanao/R11)**	**93.00**	**96.91**	**-3.91**
**Apayao (Northern Luzon/CAR)**	**106.51**	**93.98**	**12.53**
**Kalinga (Northern Luzon/CAR)**	**92.01**	**89.46**	**2.55**
**Guimaras (Western Visayas/R6)**	**102.89**	**82.65**	**20.24**
**Davao del Norte (Southern Mindanao/R11)**	**79.36**	**82.27**	**-2.91**
**Mountain Province (Northern Luzon/CAR)**	**97.59**	**81.97**	**15.62**
**Zambales (Central Luzon/R3)**	**90.93**	**81.34**	**9.59**
**Cotabato City (Southwestern Mindanao/BARMM)**	**47.45**	**78.79**	**-31.34**
**Agusan del Norte (Northeastern Mindanao/CARAGA)**	**78.85**	**77.65**	**1.20**
**South Cotabato (Central Mindanao/R12)**	**61.49**	**70.81**	**-9.32**
**Ifugao (Northern Luzon/CAR)**	**72.42**	**70.32**	**2.10**
**Pampanga (Central Luzon/R3)**	**68.65**	**68.55**	**0.10**
**Negros Occidental (Western Visayas/R6)**	**75.06**	**67.13**	**7.93**
**Surigao del Sur (Northeastern Mindanao/CARAGA)**	**70.57**	**67.02**	**3.55**
**Iloilo (Western Visayas/R6)**	**81.89**	**64.58**	**17.32**
**La Union (Northern Luzon/R1)**	**79.84**	**62.92**	**16.93**
**Surigao del Norte (Northeastern Mindanao/CARAGA)**	**66.01**	**62.47**	**3.54**
**Rizal (Southern Luzon/R4A)**	**53.65**	**61.75**	**-8.10**
**Bulacan (Central Luzon/R3)**	**59.67**	**59.84**	**-0.17**
**Ilocos Norte (Northern Luzon/R1)**	**83.15**	**58.17**	**24.99**
**Davao de Oro (Southern Mindanao/R11)**	**55.20**	**57.49**	**-2.29**
**City of Isabela (Western Mindanao/R9)**	**45.34**	**57.34**	**-12.00**
**Zamboanga del Sur (Western Mindanao/R9)**	**53.42**	**57.22**	**-3.80**
**Tarlac (Central Luzon/R3)**	**62.24**	**57.11**	**5.13**
**Oriental Mindoro (Southern Luzon/R4B)**	**56.91**	**56.55**	**0.36**
**Nueva Ecija (Central Luzon/R3)**	**60.60**	**55.42**	**5.18**
**Davao Oriental (Southern Mindanao/R11)**	**56.00**	**54.82**	**1.18**
**Laguna (Southern Luzon/R4A)**	**50.10**	**54.25**	**-4.15**
**Agusan del Sur (Northeastern Mindanao/CARAGA)**	**45.12**	**50.59**	**-5.47**
**Pangasinan (Northern Luzon/R1)**	**53.87**	**47.63**	**6.24**
**Abra (Northern Luzon/CAR)**	**62.32**	**47.37**	**14.95**
**Capiz (Western Visayas/R6)**	**58.84**	**46.68**	**12.16**
**Negros Oriental (Central Visayas/R7)**	**53.27**	**45.74**	**7.53**
**Dinagat Islands (Northeastern Mindanao/CARAGA)**	**51.58**	**43.73**	**7.85**
**Lanao del Norte (Northern Mindanao/R10)**	**34.87**	**43.02**	**-8.15**
**Antique (Western Visayas/R6)**	**53.15**	**42.03**	**11.12**
**Lanao del Sur (Southwestern Mindanao/BARMM)**	**19.76**	**40.93**	**-21.17**
**Occidental Mindoro (Southern Luzon/R4B)**	**37.01**	**39.89**	**-2.87**
**Cavite (Southern Luzon/R4A)**	**37.14**	**39.60**	**-2.46**
**Davao Occidental (Southern Mindanao/R11)**	**33.45**	**38.77**	**-5.32**
**Batangas (Southern Luzon/R4A)**	**38.13**	**36.49**	**1.65**
**Palawan (Southern Luzon/R4B)**	**31.36**	**35.99**	**-4.63**
**Ilocos Sur (Northern Luzon/R1)**	**49.27**	**35.30**	**13.98**
**Marinduque (Southern Luzon/R4B)**	**46.06**	**34.57**	**11.48**
**Aklan (Western Visayas/R6)**	**41.11**	**33.99**	**7.12**
**Bohol (Central Visayas/R7)**	**42.93**	**32.92**	**10.02**
**Misamis Oriental (Northern Mindanao/R10)**	**28.65**	**29.82**	**-1.17**
**Biliran (Eastern Visayas/R8)**	**32.45**	**28.35**	**4.11**
**Siquijor (Central Visayas/R7)**	**41.74**	**27.56**	**14.18**
**Zamboanga del Norte (Western Mindanao/R9)**	**27.82**	**27.47**	**0.35**
**Camarines Sur (Southern Luzon/R5)**	**26.72**	**26.95**	**-0.23**
**Albay (Southern Luzon/R5)**	**27.03**	**25.47**	**1.56**
**Romblon (Southern Luzon/R4B)**	**30.19**	**25.37**	**4.82**
**Zamboanga Sibugay (Western Mindanao/R9)**	**22.58**	**24.93**	**-2.35**
**Maguindanao (Southwestern Mindanao/BARMM)**	**10.73**	**24.34**	**-13.61**
**Quezon (Southern Luzon/R4A)**	**23.70**	**23.20**	**0.50**
**Basilan (Southwestern Mindanao/BARMM)**	**11.53**	**21.32**	**-9.79**
**Samar (Western Samar) (Eastern Visayas/R8)**	**19.59**	**18.84**	**0.75**
**Leyte (Eastern Visayas/R8)**	**20.69**	**18.82**	**1.87**
**Sorsogon (Southern Luzon/R5)**	**18.88**	**17.56**	**1.32**
**Cotabato (North Cotabato) (Central Mindanao/R12)**	**16.17**	**17.48**	**-1.31**
**Bukidnon (Northern Mindanao/R10)**	**14.44**	**17.00**	**-2.56**
**Northern Samar (Eastern Visayas/R8)**	**14.76**	**14.83**	**-0.07**
**Eastern Samar (Eastern Visayas/R8)**	**15.76**	**14.13**	**1.63**
**Batanes (Northern Luzon/R2)**	**21.51**	**13.63**	**7.88**
**Catanduanes (Southern Luzon/R5)**	**14.77**	**12.77**	**2.00**
**Southern Leyte (Eastern Visayas/R8)**	**16.57**	**12.30**	**4.27**
**Sultan Kudarat (Central Mindanao/R12)**	**9.63**	**11.93**	**-2.30**
**Camiguin (Northern Mindanao/R10)**	**14.02**	**11.05**	**2.97**
**Sarangani (Central Mindanao/R12)**	**8.42**	**10.79**	**-2.37**
**Camarines Norte (Southern Luzon/R5)**	**8.59**	**8.70**	**-0.11**
**Misamis Occidental (Northern Mindanao/R10)**	**9.59**	**7.72**	**1.87**
**Masbate (Southern Luzon/R5)**	**4.41**	**4.82**	**-0.41**
**Sulu (Southwestern Mindanao/BARMM)**	**1.40**	**2.63**	**-1.23**
**Tawi-Tawi (Southwestern Mindanao/BARMM)**	**1.37**	**2.22**	**-0.85**

ASMR: age-standardized mortality rate; BARMM: Bangsamoro Autonomous Region in Muslim Mindanao; CAR: Cordillera Administrative Region; CARAGA: Caraga Administrative Region (Region 13); CMR: crude mortality rate; R: administrative region.

^a^ Data are presented in descending order of age-standardized mortality.

^b^ The designations in the Province column link each province to its location in the heatmap in Fig. 1. For instance, the province of Benguet is in CAR, in the geographical region of Northern Luzon.

### COVID-19 deaths

A total of 66 566 records of COVID-19 death were available, of which 388 (0.6%) were excluded due to missing demographic data. A total of 66 178 complete records were retained for analysis and covered confirmed COVID-19 deaths that occurred between 18 January 2020 and 9 May 2023.

### Age-standardized mortality

Provinces are ranked by their ASMRs in **Table 1**. The 10 provinces with the highest COVID-19 mortality rates, in decreasing order, were Benguet (207.83 deaths/100 000 population), Cagayan, Bataan, Nueva Vizcaya, Quirino, National Capital Region, Isabela, Cebu, Aurora and Davao del Sur (96.91 deaths/100 000 population). Eight of these 10 provinces are in the Luzon island group, the northern part of the country. Moreover, four of the top five are in northern Luzon, suggesting a clustering of provinces with high ASMRs, which is particularly evident in the heatmap visualization shown in **Fig. 1**.

The 11 provinces with the lowest rates, in increasing order, were Tawi-Tawi (2.22 deaths/100 000 population, less than 1% of Benguet’s rate), Sulu, Masbate, Misamis Occidental, Camarines Norte, Sarangani, Camiguin, Sultan Kudarat, Southern Leyte, Catanduanes and Batanes (13.6/100 000 population). The geographical distribution of these provinces was more varied: Tawi-Tawi and Sulu provinces are in Southwestern Mindanao; Misamis Occidental and Camiguin are in northern Mindanao; Sarangani and Sultan Kudarat are in central Mindanao; Masbate, Camarines Norte and Catanduanes are in southern Luzon; Southern Leyte is in eastern Visayas; and Batanes is in Northern Luzon (**Fig. 1**).

### Age-specific mortality

**Fig. 2:**
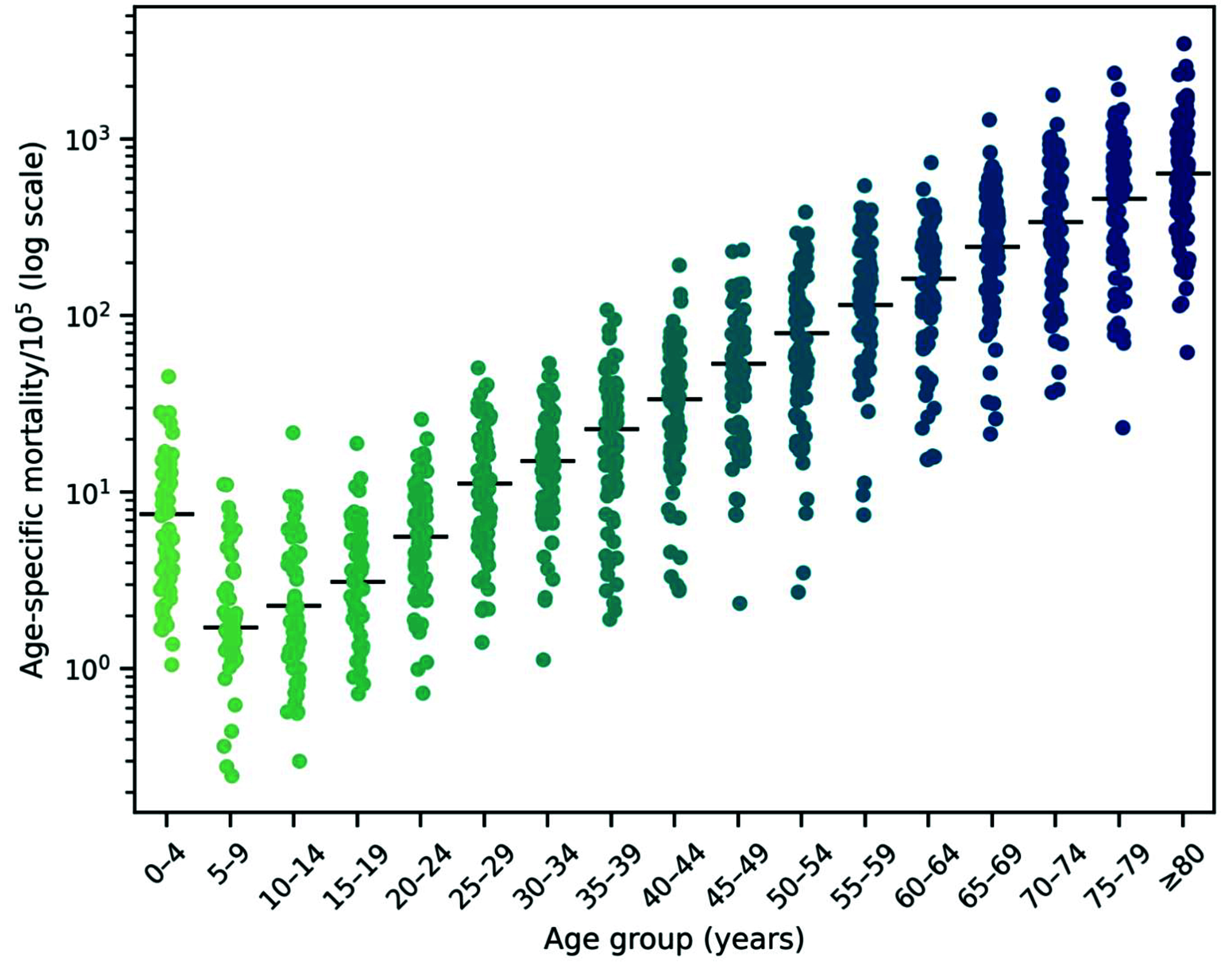
Age-specific COVID-19 mortality per 100 000 population presented as strip plots with medians, Philippines, January 2020–May 2023

Age-specific mortality is plotted in **Fig. 2** and demonstrates a J-shaped pattern. The lowest mortality was recorded in children aged 5–9 years (median: 0.53 deaths/100 000 population; IQR: 0, 1.95), but mortality rose steeply with increasing age. The highest mortality (640.90 deaths/100 000 population; IQR: 347.27, 1046.65) was recorded in the oldest age group (≥ 80 years). Interestingly, the median mortality in the youngest age group, 0–4 years (4.79 deaths/100 000 population; IQR: 2.15, 9.61), was higher than that for the adjacent age groups of 5–9, 10–14 and 15–19 years (**Supplementary Table 2**).

[**insert** Figure 2]

### Correlation analysis

Three predictor variables were found to be correlated with both the CMR and the ASMR, namely poverty incidence, hospital bed density and COVID-19 testing rate (**Table 2**, **Supplementary Table 3**). Poverty incidence was negatively correlated with the CMR and ASMR (CMR: *r_s_* = −0.55 [moderate], *P* < 0.001; and ASMR: *r_s_* = −0.34 [weak], *P* < 0.001), implying that provinces with a high incidence of poverty tended to have lower mortality from COVID-19. Conversely, hospital bed density and COVID-19 testing rates exhibited weak positive correlations with the CMR and ASMR (for hospital bed density, CMR: *r*_s_ = 0.35, *P* < 0.05 and ASMR: *r_s_* = 0.33, *P* < 0.05; for testing rates, CMR: *r_s_* = 0.44, *P* < 0.001 and ASMR: *r_s_* = 0.46, *P* < 0.001), suggesting that mortality was higher in provinces with better availability of and access to health care.

**Table 2:** Spearman’s rank correlation coefficients for associations between crude and age-standardized COVID-19 mortality and five predictor variables, Philippines, January 2020–May 2023

Variable	Mortality		Predictor variable (data year)				
	Crude	Age-standardized	Poverty incidence (2021)	Hospital bed density (2022)	Population density (2020)	% population aged ≥ 65 years (2020)	COVID-19 test rate (2020–2023)
**Crude mortality**	**1.00**	**0.97^a^**	**-0.55^a^**	**0.35^b^**	**0.10**	**0.21**	**0.44^a^**
**Age-standardized mortality**		**1.00**	**-0.49^a^**	**0.33^b^**	**0.11**	**-0.01**	**0.46^a^**
**Poverty incidence (2021)**			**1.00**	**-0.45^a^**	**-0.29^b^**	**-0.25^a^**	**-0.35^b^**
**Hospital bed density (2022)**				**1.00**	**0.32^b^**	**0.15^b^**	**0.52^a^**
**Population density (2020)**					**1.00**	**0.00**	**0.52^a^**
**% population aged ≥ 65 years (2020)**						**1.00**	**-0.01**
**COVID-19 test rate (2020–2023)**							**1.00**

^a^ *P* < 0.001.

^b^ *P* < 0.05.

Furthermore, evidence was found of correlations between several pairs of the predictor variables (**Table 2**, **Supplementary Table 3**). First, poverty incidence was weakly negatively correlated with COVID-19 testing rates (*r_s_* = −0.35, *P* < 0.05) and likewise with hospital bed density (*r_s_* = −0.45, *P* < 0.001). Second, population density showed a moderate positive correlation with COVID-19 testing rates (*r_s_* = 0.52, *P* < 0.001) and a weak positive correlation with hospital bed density (*r_s_* = 0.32, *P* < 0.05). Lastly, there was a very weak negative correlation between population density and poverty incidence (*r_s_* = −0.29, *P* < 0.05). Collectively, these pairwise correlations indicate that the more densely populated provinces with lower levels of poverty tended to have better access to, and better availability of, health-care services, including COVID-19 testing.

## Discussion

### COVID-19 mortality

Two noteworthy findings emerged from our province-level ecological analysis of COVID-19 mortality in the Philippines. First, we observed a J-shaped pattern in age-specific mortality, with the lowest rates occurring in children aged 5–9 years. Similar patterns have been seen in several other countries, including Spain, United Kingdom of Great Britain and Northern Ireland and United States of America, indicating that while children were generally at low risk of developing COVID-19 disease, newborns and children aged < 1 year experienced slightly elevated risks. (*21*) Our results add the Philippines to the list of countries where such a pattern was observed.

Second, ranking and mapping provinces by their ASMRs revealed a clustering of provinces with high COVID-19 mortality, particularly in the Northern and Central Luzon regions, but also in the National Capital Region and the Central and Western Visayas and Southern Mindanao regions. It is noteworthy that 4 of the 10 provinces with the highest rates have densely populated cities: Benguet in northern Luzon is home to Baguio City; the National Capital Region has 16 major cities; Cebu province in central Visayas has three large cities; and Davao del Sur in southern Mindanao has one major conurbation. In contrast, some of the provinces with low mortality, such as Tawi-Tawi, Sulu, Camiguin and Batanes, are remote and/or composed of small islands.

Therefore, it seems likely that geographical connectedness and population density were instrumental in driving up COVID-19 mortality in parts of the Philippines. Worldwide, COVID-19 spread faster among populations with high rates of social interaction, which is much more likely to occur in densely populated areas. Furthermore, mobility was harder to constrain in geographically connected provinces and provinces linked by established transportation routes (i.e. land or sea). It is also worth noting that in this analysis, more remote, less densely populated island provinces tended to have much lower COVID-19 mortality. However, we found no evidence of a correlation between population density and COVID-19 mortality at the provincial level, suggesting that other factors also possibly played a role.

### Predictors of COVID-19 mortality

The observed positive correlations between COVID-19 mortality and hospital bed density and between COVID-19 mortality and testing rates represent another interesting finding and indicate that mortality may have been influenced by the availability of health-care services and capacity for disease surveillance. During the pandemic, hospitals and testing centres served as data collection and reporting sites. Thus, it follows that COVID-19 deaths that occurred in hospitals or health-care facilities with RT–PCR testing capacity were more likely to have been recorded than those that occurred in provinces with fewer resources, where a higher percentage of deaths may have occurred outside health-care facilities and were not confirmed as COVID-19 deaths.

The study also found a negative correlation between COVID-19 mortality and poverty. This relationship could be interpreted in terms of immunity, but this would imply that low-income communities had greater immunity against COVID-19. A wealth of published evidence suggests that this is unlikely. Numerous studies have consistently shown that COVID-19 disproportionately affected low-income populations, (*13*) including one study from Sweden that found having a lower socioeconomic status was linked to a higher risk of COVID-19 mortality. (*14*) Explanatory factors implicated in this relationship have included overcrowding, employment that did not allow people to work from home, financial uncertainty, reduced health-care access, and a higher burden of undiagnosed or untreated comorbidities. Evidence of negative correlations between the incidence of poverty and hospital bed density – and between poverty and COVID-19 testing – add further weight to the case against immunity.

Instead, we hypothesize that a more likely explanation for the observed correlations stems from what is widely referred to as the streetlight effect, a phenomenon whereby the number of cases is higher in populations with better surveillance. (*22*) Support for our hypothesis comes from an earlier study, conducted in 2020, in the Western Visayas region. (*23*) The authors of the Western Visayas study analysed data about testing, infection and contact tracing and found that the highest number of cases was recorded in Iloilo City, which housed the only regional COVID-19 testing facility at that time.

We further hypothesize that in the Philippines, the streetlight effect may have been exacerbated by patterns of health-seeking behaviour. Cross-sectional surveys conducted in low-income households in the Philippines, initially from 20 February to 13 March 2020, and again from 12 November to 12 December 2020, showed an increase in the practice of preventive measures (e.g. avoiding crowded places, handwashing, using disinfectants and mask-wearing). According to these repeated surveys, there was also a drop in the intention to seek care from public hospitals when exhibiting symptoms of COVID-19 and an increase in self-medication with stored medications and antibiotics. (*24*) Among the reasons cited by participants to explain their low levels of seeking health care during the pandemic were concerns about discrimination at public health-care facilities due to their socioeconomic status, high transportation costs due to mobility restrictions, stigma around contracting the virus and mistrust in control protocols. (*24*) Therefore, it seems plausible to assume that low-income households, which already had disproportionately poorer access to health care before the pandemic, were even less likely to seek treatment for COVID-19 symptoms and less likely to have been tested. Consequently, fewer deaths would have been attributed to COVID-19, further depressing rates of COVID-19 mortality relative to wealthier provinces.

### The case of Benguet

Benguet, with 207.83 deaths/100 000 population, had by far the highest computed ASMR, but it also ranked high in terms of testing capacity and coverage of contact tracing. Indeed, in 2020, Baguio City and the province of Benguet overall were commended by WHO for their exceptional COVID-19 pandemic response, which included surveillance. (*25*) It is highly likely that this level of response meant that COVID-19 deaths were more likely to be recorded in Benguet than in other provinces. However, this is not to say that high rates of testing and contact tracing were the only factors that contributed to Benguet’s extremely high COVID-19 mortality.

A high population density was likely a contributory factor, given that Benguet is home to Baguio City, one of the more densely populated cities in the Philippines. Another possible factor is Benguet’s climate. Baguio City has a mean annual temperature of 18 °C, making it the coldest city in the Philippines. (*26*) Several ecological studies (*27*-*29*) have reported associations between temperature, humidity and COVID-19 mortality, in which areas with colder and more humid weather – where people typically spend more time indoors – have consistently recorded more cases and deaths.

### Limitations and recommendations for future work

Ecological studies have inherent limitations, including an inability to account for local dynamics within a province. A fuller understanding of the drivers of COVID-19 mortality and the effectiveness of public health measures and interventions would require closer, more detailed investigation of individual provinces. Nevertheless, this analysis, by identifying provinces with very high and very low mortality, represents a useful first step. Moreover, Benguet has emerged as a good candidate for further epidemiological investigation, given its high mortality and unique combination of drivers of COVID-19 mortality, including a pronounced streetlight effect.

We were unable to include several important variables in our analysis, namely government policies, such as travel restrictions and lockdowns, climate variables, comorbidities and immunization status. Nor were we able to assess the impact of temporality; this is likely an important consideration for certain factors, such as testing rates, which will have changed through time. To overcome some of these shortcomings, future investigations should consider using alternative study designs, such as multivariate correlation analysis, multivariate regression and causal inference. Alternative means for estimating COVID-19 mortality, such as excess deaths, might also be useful. (*30*)

### Conclusions

This study is the first to present a province-level analysis of COVID-19 mortality in the Philippines and, in addition to identifying provinces with high and low rates, found evidence of clustering of provinces with high rates. The study also found evidence of a so-called streetlight effect and concluded that the rankings of mortality, at least in part, are influenced by unequal surveillance capacity among provinces. We recommend further investigation of local dynamics within individual provinces to better understand the drivers of mortality and the impact of the interventions and control measures that were implemented during the pandemic to reduce mortality. These findings pave the way for future work by identifying some of the factors that will need to be considered and more closely investigated to get a clearer picture of what happened during the pandemic and to inform decision-making for future pandemic preparedness and response, and thereby safeguard millions of lives in communities across the Philippines.
