# Supplementary material for: COVID-19 mortality in the Philippines: province-level ecological analysis, 2020–2023
Source: Western Pac Surveill Response J. 2026 Mar 25;17(1):1–12. doi: 10.5365/wpsar.2026.17.1.1128 (PMC13056467; doi:10.5365/wpsar.2026.17.1.1128)
Supplement: Supplementary file 1 [file wpsar-17-1128-s001.pdf]

Supplementary Fig. 1. **Pairwise correlations between crude mortality rates (CMRs), age-standardized mortality rates (ASMRs), poverty incidence, population density, number of hospital beds per 100 000 population, percentage of population aged ≥65 years and number of COVID-19 tests per 100 000 population, Philippines, January 2020–May 2023**

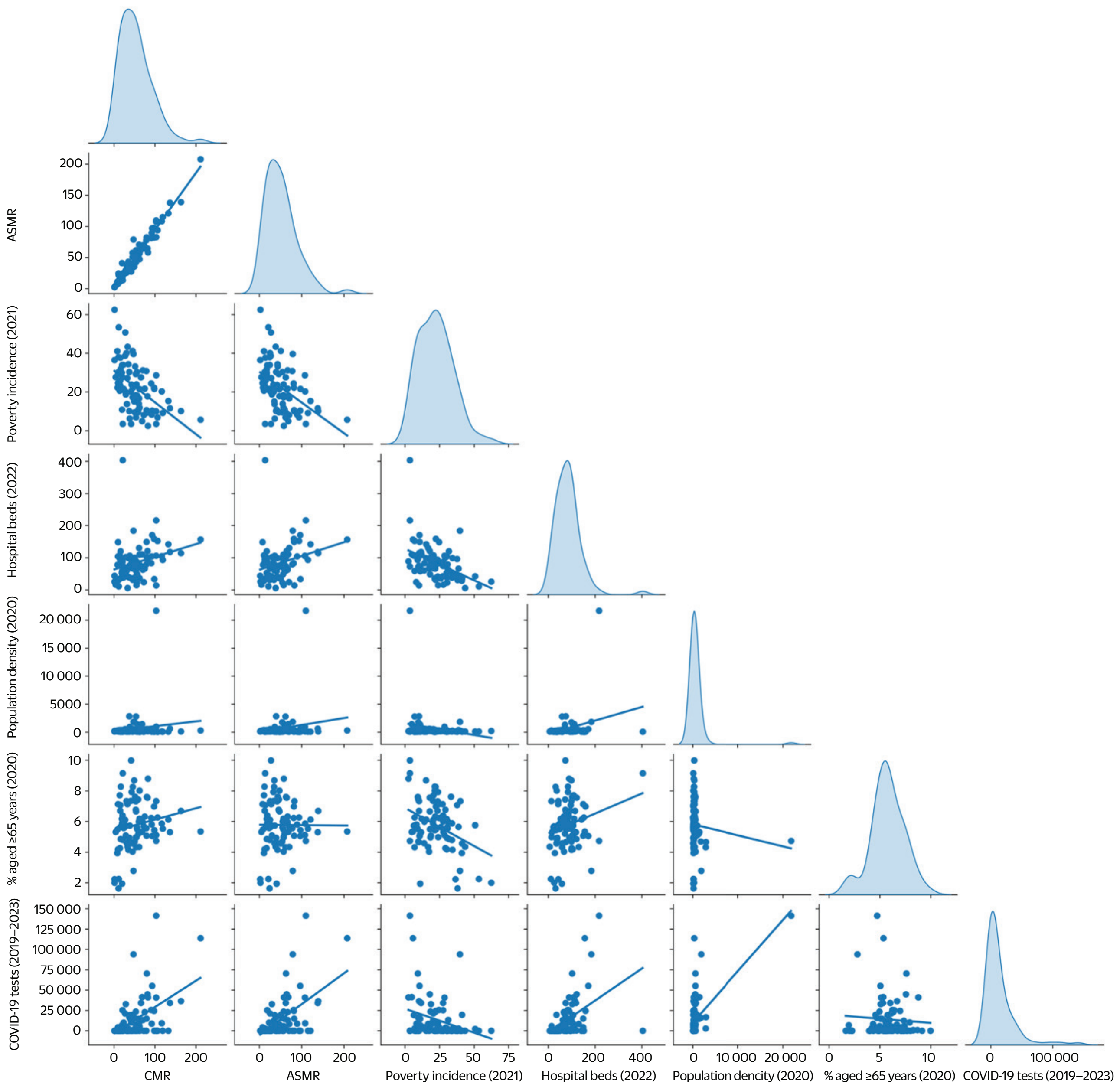

Supplementary Table 1. **Mean and median crude and age-standardized province-level COVID-19 mortality per 100 000 population, Philippines, January 2020–May 2023**

Variable	Mean	Standard deviation	Minimum	25%	Median	75%	Maximum
Crude mortality	53.41	38.90	1.37	22.32	48.36	73.08	211.03
Age-standardized mortality	51.31	36.64	2.22	24.78	44.73	67.48	207.83
Difference (crude – age-standardized)	2.10	8.86	-31.34	-2.35	1.26	7.22	24.99

Supplementary Table 2. **Mean and median province-level COVID-19 age-specific mortality per 100 000 population, Philippines, January 2020–May 2023**

Age group (years)	Mean	Standard deviation	Minimum	25%	Median	75%	Maximum
0–4	7.22	7.81	0.00	2.15	4.79	9.61	45.07
5–9	1.51	2.34	0.00	0.00	0.53	1.95	11.06
10–14	2.14	3.10	0.00	0.00	1.31	2.42	21.65
15–19	2.91	3.36	0.00	0.00	1.95	4.47	18.85
20–24	6.08	4.94	0.00	2.49	5.12	8.42	25.79
25–29	11.74	10.71	0.00	4.77	8.67	16.50	50.44
30–34	15.78	11.82	0.00	7.84	14.35	21.51	53.59
35–39	24.07	21.55	0.00	7.40	20.70	33.98	107.36
40–44	35.44	31.63	0.00	15.24	29.84	46.04	192.31
45–49	58.63	48.08	0.00	23.96	46.23	69.63	234.53
50–54	92.27	75.86	0.00	39.89	64.83	120.15	384.16
55–59	145.48	104.88	0.00	76.67	124.86	183.13	542.28
60–64	195.86	136.76	0.00	92.27	187.60	271.82	732.91
65–69	305.77	206.24	0.00	157.96	284.43	391.84	1279.52
70–74	435.95	311.59	36.57	194.27	364.22	593.63	1769.76
75–79	545.14	442.88	0.00	211.89	472.67	736.99	2350.30
≥80	792.22	640.48	0.00	347.27	640.90	1046.65	3453.26

Supplementary Table 3. ***P* values for the correlation analyses between crude and age-standardized COVID-19 mortality and five predictor variables, Philippines, January 2020–May 2023**

Variable	Mortality		Predictor variable (data year)				
	Crude	Age-standardized	Poverty incidence (2021)	Hospital bed density (2022)	Population density (2020)	% population aged ≥65 years (2020)	COVID-19 test rate (2020–2023)
Crude mortality	<0.001	<0.001	<0.001	<0.05	0.204	0.109	<0.001
Age-standardized mortality	<0.001	<0.001	<0.001	<0.05	0.081	0.962	<0.001
Poverty incidence (2021)	<0.001	<0.001	<0.001	<0.001	<0.05	<0.001	<0.05
Hospital bed density (2022)	<0.05	<0.05	<0.001	<0.001	<0.05	<0.05	<0.001
Population density (2020)	0.204	0.081	<0.05	<0.05	<0.001	0.338	<0.001
% population aged ≥65 years (2020)	0.109	0.962	<0.001	<0.05	0.338	<0.001	0.543
COVID-19 test rate (2020–2023)	<0.001	<0.001	<0.05	<0.001	<0.001	0.543	<0.001
